# Supplementary material for: Controlling gene expression timing through gene regulatory architecture
Source: PLoS Comput Biol. 2022 Jan 18;18(1):e1009745. doi: 10.1371/journal.pcbi.1009745 (PMC8797265; doi:10.1371/journal.pcbi.1009745)
Supplement: Attachment — Submitted filename: Timing_Plos_review.pdf [file pcbi.1009745.s012.pdf]

We thank the reviewers and editors for the thoughtful assessment of our manuscript. We have amended the manuscript, added additional figures in supplementary, added references and modified figures to address all the critiques raised by the reviewers. We believe the manuscript is significantly stronger now thanks to the careful comments and suggestions from the reviewers.

We have worked particularly hard to address one criticism raised by all three referees (in one way or another) regarding the generalizability of our findings. Although the manuscript featured bacterial timescales, rates and models for protein degradation, the results do not depend on this choice. We have expanded the manuscript in several areas to demonstrate the generalizability of our results to be more consistent with simple Eukaryotic models. Specifically, we show that neither the mode and rates of protein degradation nor the mRNA lifetimes impact our findings in a meaningful way. As a result, we have attempted to remove language in the manuscript implying that our results are specific to bacterial physiology and to stress the broad generalizability of our findings. We believe this change alone has significantly improved the manuscript.

Furthermore, we address each specific comment from the referees below. Changes are highlighted in red in the revised manuscript. Line numbers correspond to that from the revised manuscript.

Reviewer #1: Summary:
In “Controlling gene expression timing through gene regulatory architecture”, Ali and Brewster explore qualities of transcription factor (TF) circuits that tune timescales of activation or deactivation of gene expression states. Their work adds to a field that often focuses on steady-state characteristics of gene expression.

Ali and Brewster ask how the parameters of one of the simplest circuit motifs (a single node with feedback) influence the speed at which the circuit reaches steady state expression (SSE). The authors chose a well-precedented framework for the investigation by applying a stochastic simulation algorithm (Gillespie algorithm). This allows them to evaluate single-cell trajectories, getting both fine detail as well as characterizing bulk population dynamics. Using first passage time (FPT) as a metric for the timescale at which a circuit progresses to a new equilibrium, they modulate binding affinity, decoy sites, and auto-strength, and examine the resulting effects on FPT. With these analyses, the authors make several findings that may help guide analysis of native gene circuits and design of gene circuits with desired timing properties.

The dynamics of regulatory circuits is a timely and important problem, particularly in designing more complex circuits that may interact with dynamic signals (Gerardin et al., Cell Systems, 2019; Bashor et al., Science, 2019). In addition to analyzing bulk population timing response with the summary statistic mean FPT (MFPT), Ali and Brewster also use the

stochastic simulation to illustrate variance among single cell trajectories. The two analyses together find that MFPT can be achieved with different noise levels, enabling design strategies that can tune both variance and average timing.

Separately, the use of decoys has recently taken focus as a strategy for tuning steady-state expression levels (Wan et al., Nat Commun, 2020; Wang et al., Nucleic Acids Res, 2021), and have played an important role in controlling timing aspects in dynamics systems as well (Potvin-Trottier et al., Nature, 2016; Henningsen et al., ACS Synth Biol, 2020). Here, Ali and Brewster quantitatively evaluate the role of decoys on timing of a single node with feedback. They find an impactful result in that decoys may increase FPT tremendously up to 100s of generations, such that the system might not actually reach SSE.

Finally, the authors examine the effects on target gene regulation alongside auto-regulation. They find a large space of regulatory timing that can be produced by single node feedback. The work indicates that auto-regulatory architecture is fundamental in timing of the target.

Overall, the paper succeeds in exploring the effects of TF circuit parameters on the timescales of the system and its targets. The results inspire new ideas about how to analyze and design TF circuits with desired timing properties. Specific comments and questions that could enhance the work are enumerated below.

Specific questions and comments:

1. There are several directly relevant works that should be cited as state-of-the-art for how the temporal characteristics of gene circuits have been explored and how they are currently designed.

a. Specifically, in “The Design Principles of Biochemical Timers: Circuits that Discriminate between Transient and Sustained Stimulation” by Gerardin, Reddy, and Lim, basic network motifs (including 1-node feedback architectures) are extensively characterized with focus on timing of responses. These characteristics include “trigger time,” which is analogous to FPT, as well as a host of other qualities.

b. Additionally, in “Complex signal processing in synthetic gene circuits using cooperative regulatory assemblies” by Bashor et al., the same circuit parameters (regulatory architecture and binding affinity, i.e., koff) are examined across a large space of configurations to generate a space of “activation/deactivation half-times,” which is again analogous to FPT. In addition to the computational model space examined, the work supports the predictions with synthetic circuits in living cells.

A fundamental difference among the papers mentioned and the present Ali and Brewster work is the kind of model used. The application of a stochastic simulation model in the present work enables different kinds of analysis such as a variation in timing among single cells, “noise”.

We thank the reviewer for suggesting these very relevant references, we have added a citation to them in the introduction where appropriate.

2. For the power-law relationship between peak MFPT and auto-strength (alpha) during auto-activation, can the authors comment further on (A) why this power law is generated, (B) why this power law does not exist in auto-repression, and (C) why the exponent changes with MPFT threshold?

This is a question that we have spent significant time and energy exploring. At the moment we do not have answers for these questions but the origin of this power law is something we are continuing to explore. We managed to get an analytical solution for response time in a simple deterministic system, analogous to MFPT, and show that the analytical solution is consistent with the findings. Unfortunately, we could not solve it to find the maxima of this analytical form and show the power-law dependence.

3. The authors analyze a large range of decoys that can be used to tune regulatory timing. The plots in Figure 4 look at hundreds to thousands of decoys used, and the authors suggest that at high decoy numbers, cells can generate an interesting phenomenon where they may never reach steady state. What is the context at which these numbers are reasonable to consider? For instance, for a particular native transcription factor, are hundreds to thousands of sites present in a genome? Or if designing synthetic circuits, is it reasonable to deliver such a large payload with high repetitiveness?

Of course, the reasonable range of decoy numbers depends on the specific TF. For instance, highly shared TFs like CRP have several hundreds of cites per genome copy and depending on growth rates, 8 or more copies of the genome may exist, at least partially, in each cell. This problem is likely worse in Eukaryotes and although we have based on our study with bacteria in mind, we have worked to generalize the approach of the study (for instance, we now show our results are general regardless of degradation model as well as protein or mRNA degradation rates).

4. The few examples of noise vs koff show that it is monotonically increasing (auto-activation) or decreasing (auto-repressing) with increasing koff. It is unclear if this holds for all or a large region of the parameter space explored. Does the koff at the local extrema in MFPT always generate some intermediate noise level?

In the parameter regime probed, we never get non-monotonic behavior for CV as a function of k_off for both auto-activation and auto-repression. As a result, the noise for local extrema in MFPT is always in the intermediate range of noise level. We have expanded the section on noise.

5. The authors make an interesting comment that the FPT distribution is bimodal in strong auto-repression. Out of curiosity, is there any idea as to why this occurs? Is this largely beyond physiological ranges?

We see bimodality only when the TF binding rate is very strong (akin or perhaps stronger than the ideal LacI operator site Oid, for reference). It is unlikely that this is common in normal physiology; the parameter ranges are feasible but not typical.

6. In examining expression timing of a target gene, the authors chose to include auto-repressors that activate targets and auto-activators that repress targets. Interestingly, it is in these cases that the target regulation is sped up compared to constitutive targets. How common are these regulatory architectures in nature and is there a precedent for designing them in synthetic contexts?

We have added a panel to figure 1 that quantifies this for *E. coli*. We now show 70 autoregulatory TFs (both positive and negative) along with their number of positive and negative targets. Surprisingly, we see negatively autoregulated TFs with activated targets and vice versa.

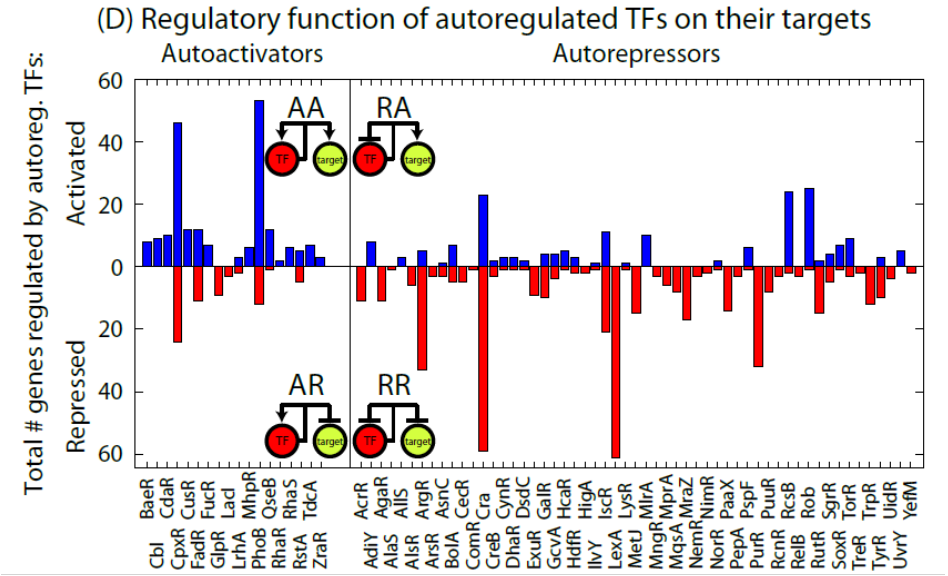

7. The authors take generation time as the dominant force of decay for proteins. In a stochastic model, how impactful would it be to consider decay as a large synchronous event (i.e., cell division) instead of spontaneous degradation of individual molecules over time (as is currently assumed)?

The reviewer has raised a very interesting point. We find that modeling cell division as the main process for cell content dilution does not alter any of our findings. We explore the effect of explicit cell division along with very slow protein degradation (~5-10 hrs lifetime; setting protein degradation to zero also reproduces the features) in a supplement section. Particularly, we focus on the effect of koff rate of the TF gene on MFPT keeping the gene copy number fixed, however, incorporating a simple scheme of gene duplication at a certain time point of cell cycle (e.g. at 0.95 x cell-cycle) gives similar results.

We incorporate cell division in the reaction scheme where the cell divides after a certain time interval, \tau_c, drawn from a gaussian with a given mean and standard deviation every time a cell divides. The cell contents are partitioned binomially between the daughter cells after every division. For simplicity, we assume a single gene copy throughout the cell cycle. Below is the figure generated using a 50 min cell cycle when off-rate is tuned for autoregulated genes. We find non-monotonic behavior of MFPT as a function of off-rate as mentioned in the main manuscript where the degradation of protein is random with a half-life of one cell-cycle. We also find a power-law relationship between peak MFPT and regulatory strength (alpha) as well as off rate at peak vs alpha for auto-activated genes.
We must mention that the details of cell division such as how the cell contents are partitioned (binomially or equal partition) or with and without gene duplication do not alter our inferences at least for a simple scheme.

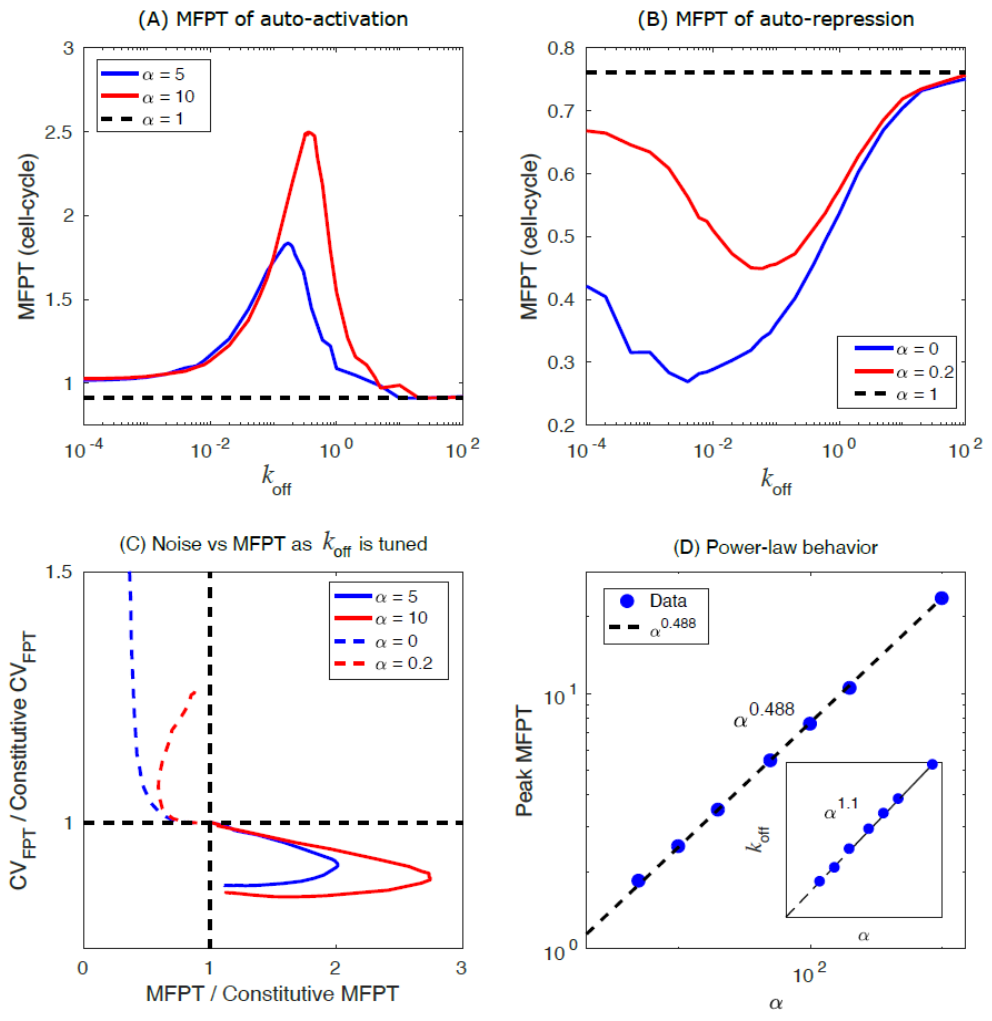

8. The caption on Figure 5 has mixed up the panel reference. It refers to (C) as (A) and (A-B) as (B-C).

Thank you for pointing out the mismatch. We have now fixed it to correctly read the panel references.

9. Should lines 136-139 say that SSE decreases as binding affinity decreases (i.e., k_off increases)?

k_off and binding affinity are inversely related, i.e., higher affinity corresponds to low off rate and vice versa. This is mentioned in the model section,
"The TF binding affinity is controlled through the unbinding rate (koff), which is related to the sequence specificity of the binding site; higher affinity means lower unbinding rate and vice versa [49]."

10. CV should be defined the first time it is used in the paper in the “Results” section, subheading “Model”, line 91.

We have modified the sentence to read “the coefficient of variation (CV) ”

11. In supplemental Figure S3, panel (B), the title reads ‘locaiton’ instead of ‘location’. Also in Fig S3, are open circles auto-repressing (alpha < 1) and the caption has it reversed?

Figure S3, panel B has been corrected. The open circles are for auto-repression. We have fixed this typo.

Reviewer #2
The authors develop and analyse kinetic Monte Carlo simulations to investigate how the timing of gene expression in auto-regulated genes (activation or repression) is affected by TF binding affinity and competition for TFs (via decoy sites).
Most of the manuscript is a nice reading, with simple concepts and approaches. I am surprised no one thought to investigate auto-regulated circuits with a simple computational approach such as this one (this is not to be intended as a negative comment).

I would suggest the authors to better introduce the literature on the topic, as it is already partly done in the introduction, and in particular emphasise more the analogies, differences and breakthroughs of their works. For instance, what are the differences with, e.g. ref.[27]? To my understanding the most relevant feature introduced in Ali and Brewster is the presence of a limited number of TFs, which allows them to study the effects of the competition with other binding sites.

1. I understand that taking a threshold that is relative to the steady-state value is something consistent with the literature, but would the results change by taking an absolute value of expression as a threshold? I imagine that in many regulatory systems it is the concentration of TFs determining the expression (or not) of a product, not the relative expression with respect a steady-state value.

This is a very important point. The choice to take a relative threshold mostly eliminates the promoter strength as a scaling parameter and highlights the role of regulation of that promoter. Activating a promoter of a set rate will always make it reach a set level of expression faster than repressing it (in fact, it might not reach that level at all if you add repression). Using a relative threshold is a way to normalize two different scenarios to measure the dynamics of a promoter that reaches a set level of expression. As an example, the famous result that autorepression “speeds up” the response of a gene is only true if you

take care to normalize the promoter such that the levels are the same (or to use a threshold).

2. I have the impression that sometimes the authors could dig more to rationalise their Results.
For instance, what is the origin of the power-law dependences in Fig.3?
The CV is discussed in a few lines only, and panels (C) and (F) of Figure 5 are difficult to understand and could be introduced better in the text.

We re-worded and expanded this section and attempted to expand our discussion of the results. We have added further discussion of the CV (thanks, in part to a helpful suggestion in your comment #5 below). As for the origin of the power-law dependencies, we have spent considerable time on this and still don't have a satisfactory explanation. We have tried to answer through toy models or exact solutions but have not had much success. Typically, power-law behaviors are the result of a stochastic process giving rise to long-tailed distributions, however here we actually see this behavior in both the stochastic and deterministic treatment, so we haven't had success with "typical approaches".

3. An attempt to make an analytical theory on a few aspects could have been tried (even just a few back to the envelope estimates).
We have added a section "Toy-model" in the supplementary where we describe a simplified deterministic model of autoregulation. The model can be solved exactly to get an analytical expression for response time (time to reach a certain threshold, analogous to MFPT). The analytical solution recapitulates all the features for an autotregulatory gene. However, we could not get a simplified analytical form for peak response time, even for this simplified model.

4. Furthermore, discussing the biological consequences of the results could help to increase the impact and resonance of this paper. I do not know if there are available datasets that could be analysed, but in case I would encourage the authors to do so.

We have expanded the discussion with the intention of further stressing the important implications of these results. We have also worked hard to demonstrate that our results are generalizable beyond only applying for parameterizations consistent with bacterial systems. Unfortunately, there are not any obvious data sets with the rigor we would require.

5. The manuscript sometimes lacks of precision. For instance it seems to me that the caption in Fig.3 does not correspond to what is represented in the figure (insets), and in the caption of Fig.5 the panels have been inverted.

We apologize for the incorrect caption in Fig. 5. Now, we have fixed the captions to correctly read the panels. However, we could not find the inaccuracies in Fig 3, we have rewritten the caption slightly in case our word choices were confusing.

In the same would be helpful to plot the ratio CV/CV(constitutive)?

This is a fantastic suggestion. We have modified Fig. 5 C and F to show scaled CV as a function of scaled MFPT. The scaled figure clearly shows a continuum in CV from auto-activated to auto-repressed genes. As such we find that when k_{off} is tuned the noise in auto-repressed genes is higher than that of auto-activated genes. The story is reversed when the decoy number is tuned (the CV and MFPT is scaled by CV and MFPT of zero decoy motif).

What is the role (and value) of α_t (introduced in Fig.1) in Figure 6?

We should have mentioned that since there is no feedback of alpha_t in the TF production, it has no effect on the MFPT of TF or target genes. We generally fix it such that the target expression is of the order of hundreds of molecules. We mention this now in the manuscript where applicable.

6. The authors should include more details on their method, expanding the dedicated section to facilitate the reproduction of their results (see also the comment on α_t above). For instance, how is the 50% threshold calculated? Is it the analytical estimate or the value of the (mean) numerical steady-state?

Alpha_t: we apologize for not mentioning how alpha_t affects MFPT and now explicitly mention this in the method section (also see comment 5).

Threshold calculation: In the Materials and methods section, we explicitly mention that we first estimate steady state expression using stochastic simulation and then use this as input to compute mean first passage time.

Sentences like " In this plot, the number of total TFs is kept constant by adjusting the translation rate to counteract the effect of changing koff" about Figure 6 are not easily understandable without a deeper discussion.

We now clarify the need to adjust the translation rate in the manuscript.

“Tuning the off rate of the target changes the free TF availability. For instance, if the TF binds to the target gene weakly, more TFs will be available to regulate the TF gene itself and vice versa. This effect is more pronounced at low TF copy number\cite{Ali2020}.”

7. I would suggest to edit and expand the last 2 sections of the paper (from page 9), the explanation and discussion of the results.

Both sections are now expanded significantly. Changes are marked in red.

MINOR

- In the Author Summary: "which dictate" --> "which dictates"?; "pose a problem" --> "poses a problem"?

Fixed, thank you.

- Top page 2, "TF" already introduced on line 2.
Corrected.

- Line 16: "However, despite the prevalence of this regulatory motif in natural genetic circuits and the importance for efficient transcriptional programs, we can not predict or design temporal gene expression patterns from this motif based on the regulatory sequence of the regulated genes." Why? I am not sure I understand. Can the author explain better?

We have reworded this sentence to hopefully clarify its intentions

- "Importantly, the MFPT is a single-cell measurement of response time; as opposed to measuring how long the bulk takes to reach a certain expression level on average." Can the authors further develop this sentence?

This is simply a statement that averaging the time it takes for individual trajectories to reach a threshold (MFPT) is different from averaging all of those trajectories first and measuring how long it takes to reach that same threshold (bulk measurement). We have attempted to clarify this in the quoted section.

- Why only changes in k_{off} (and not on k_{on}) are considered? Is this because the binding events can be assumed to be always diffusion limited?

The reviewer is correct. We assume the on-rate is diffusion-limited and is constant whereas the off-rate controls the binding affinity. This is a typical assumption for modeling TF dynamics. We now specifically mention this in the Model section along with references.

- In the references, 'jacob'-->'Jacob'

We have fixed this.

Reviewer #3
In this work the authors use stochastic simulations to analyse the timing of gene activation or repression for the “single-input module” network motif, where a TF regulates itself and a target gene. They characterise the mean and the variance of the first passage time (FPT) distribution for the autoregulated TF alone and a target gene, for activating and repressive TF function. They focus on how these quantities depend on the TF unbinding rate (affinity) and the number of competing binding sites on the genome. For the autoregulated gene alone, they find a nonmonotonic relationship between affinity and MFPT and between number of competing sites and MFPT. The relationship between the noise of the timing and these two parameters depends on the type of regulation. Finally, they show that the MFPT of the TF gene determines that of a target gene.

This is a relevant topic and the methodology is correct to the best of our knowledge. The scope of the study is limited to a specific motif in a bacterial setting, which allows the authors to systematically analyse timing in this system. However, the limited scope isn’t always made clear and generalization could be improved, as suggested below. There are also some points that should be clarified prior to publication.

MAJOR COMMENTS

1) Protein degradation rate. The introduction makes reference to both bacteria and eukaryotic cells (lines 20-30). However, the authors assume a bacterial setting, where the protein degradation rate is determined by cell division. This choice should be made clearly and early in the introduction. To improve generalizability, the authors could include a more thorough examination of how the results depend on the protein degradation rate, since it doesn’t seem like the simulations explicitly incorporate the cell division process.

This is a great suggestion. Instinctively, because we work experimentally in bacteria, we chose typical bacterial rates. However, we have found that none of our results or findings are sensitive to these choices. We have now expanded our examination of the dependence of degradation rate. In particular we have added a section specifically using a cell division event driven degradation model (cell contents, protein and mRNA, are partitioned between

mother and daughter binomially every cell division). Furthermore, we have expanded our range of degradation rates in Figure (S2, S6 and S9 to include faster protein degradation and slower mRNA degradation (as is more typical of Eukaryotes). In all cases our results are not qualitatively different.

It could also be useful to examine if/how the results depend on the mRNA degradation rate. See also comment 2c.

We now discuss the effect of mRNA degradation in the main text and also added two supplementary figures. Broadly, the features such as non-monotonic behavior and power-law remain the same. The peak MFPT is higher for low mRNA degradation for a given cell-division rate and exhibits the same power-law exponent. The k_off value at the peak versus regulatory strength (\alpha) collapses onto a single curve when scaled by the constitutive TF expression (Y_constitutive = r_0 b / gamma gamma_m).

2) Fig. 2 and associated text.

a) How is TF occupancy calculated?

We determine the steady state TF-occupancy from simulation in the same way as steady state protein number distribution is calculated. At the end of each iteration of the simulation we record whether the TF-promoter is occupied or not and assign a value 1 (occupied) or 0 (unoccupied). We then take the average of this value over ~10^5 iterations. This average value is the TF-occupancy.

We specify this in the method section.

b) How is R defined/calculated? ("TF production rate from a single TF"- line 127)

We use the quantity R to explain the non-monotonic behavior in a simple approach. Here, R is basically the mRNA production rate in a setting where there is only one TF molecule present and can be written, for deterministic model, as

R = r_0 x P_free + r x P_occupied
= r_0 + (r - r_0) x P_occupied
= r_0 + (r - r_0) x k_on / (k_on+koff)

P_free and P_occupied are the probabilities that the promoter is free or occupied and is given by
P_free = k_off / (n_TF x Kon + Koff), P_occupied = n_TF x Kon / (n_TF x Kon + Koff).
R is then calculated by setting n_TF = 1.
We now mention it in the manuscript.

3) It would be interesting to compare the results of Fig. 2 to a deterministic model of autoregulation. Would the same conclusions hold for the non monotonic relationship between koff and MFPT? Is there any additional insight gained from the stochastic simulations for the MFPT?

We now include a supplementary section on deterministic model and show that the results are consistent. Both the stochastic and deterministic models show a non-monotonic relationship between koff vs MFPT as well as decoys vs MFPT. We would like to clarify that in principle, the MFPT from the stochastic model and the time to reach a certain threshold for the average expression from ODEs are fundamentally different quantities. We chose to use the stochastic model for the following reasons:

1) The steady state expression can vary significantly between deterministic and stochastic models as shown in our previous paper (Ali et al., eLife 2020;9:e56517). Since, in our manuscript, the timing is computed based on steady state expressions we chose the stochastic model.
2) Additionally, an added advantage of using a stochastic approach is that noise in timing can also be computed.

To further emphasize this, we now include a supplementary section with a figure (figure S8) to compare the deterministic and stochastic MFPT curves. The figure shows non-monotonic behaviors, however, the quantitative values of MFPT can be higher or lower for the deterministic model compared to the stochastic one. The difference is more prominent when decoys are tuned.

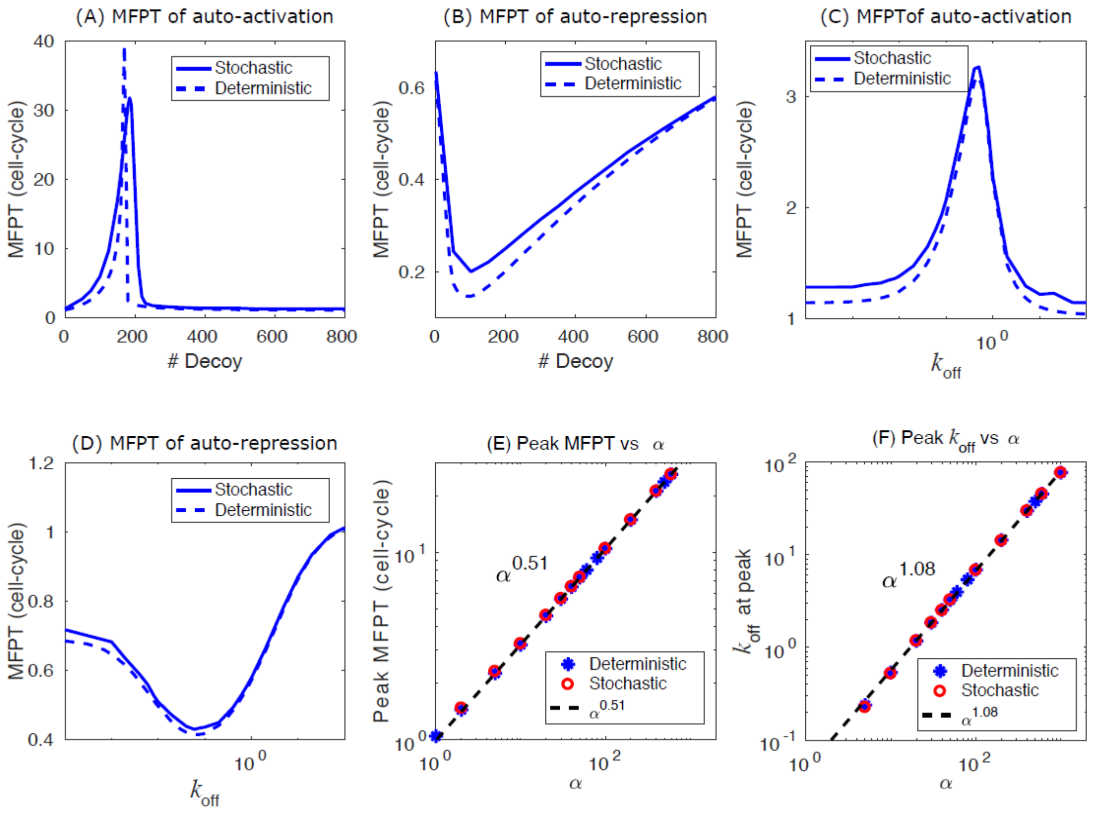

c) Fig. S2 shows the nonmonotonic behaviour as a function of the protein degradation rate. What is the influence of the mRNA degradation rate? Also, it would help to reference panels C and D of this figure near the discussion of Fig.2 in the main text.

We have added an additional figure (Figure S2) to show the effect of mRNA degradation rate. Also, we refer to these figures in the main text.

4) The analysis presented in Fig. 6 is difficult to follow.
Please clarify what is exactly being done in panels A and B.
Lines 260-261): “In this plot, the number of total TFs is kept constant by adjusting the translation rate to counteract the effect of changing koff. “ Does this mean that in panels A and B koff has the same value for both the autoregulated TF and the target TF, and it changes for both? (if it were only changing for the target, there wouldn´t be need for adjustment. But then wel don´t understand the last paragraph “We further explore the expression timing relationship when the binding sequence for TF and target genes are exactly identical, which means the binding affinity of TFs being identical for both TF and target gene.”) What is the value of alpha for the target gene in panels A, B, C?

We apologize for the typo, it should be “changing k_{off,t}” and keeping k_off constant . We corrected this typo and now clarify why there is a need of adjusting the translation rate of TF gene to keep TF number constant. The reason is that the off-rate of the target gene changes the availability of free TF, which can alter the TF production. As a result for an auto-activating TF gene, when the TF binds to the target gene very strongly, we expect a lower number of TF at steady state compared to when it binds to the target gene weakly. The opposite is true when the TF gene is auto-repressing.

We have now mentioned the values of alpha_t for the genes in panels A-C in Fig. 6. We also mention that since there is no feedback of the target protein, the MFPT of the target gene doesn’t depend on the translation rates for the same gene. However, the noise can be affected by translation/transcription rates.

Lines 272-273:” The MFPT curve of a target gene regulated by an auto-repressed TF gene is always greater than the MFPT of a target gene under the control of an auto-activated TF gene.” Please specify that this holds only for target repression.

Thanks for pointing it out. We have corrected the statement to “The MFPT curve of a target gene repressed by ”

Line 290. “It further provides us with a tool to identify the mode of TF-gene” do the authors mean TF-gene autoregulation?

Yes, the mode of TF-gene refers to how the TF gene is regulated (auto-activation or auto-repression). To clarify, we have modified the sentence to read “ identify whether the TF-gene is auto-activated or auto-repressed.”

What happens to the timing noise properties in the situation of Fig. 6? Does it just propagate trivially from the input to the target, or are there parameter combinations that reduce noise for the target compared to the input?

Interestingly, the behavior is not trivial. In case of target activation, usually we find that the noise of target gene is always less than that of the TF gene. However, when the target gene is repressed, the noise can be higher or lower compared to the noise of TF gene. We now mention this in the manuscript along with a supplementary figure.

5) The discussion and conclusions are quite short. The implications of the work are made clear, but the relationship to other work in the field on how autoactivation and autorepression change the response time of the system isn’t adequately addressed.

We have expanded the discussion significantly. We now further address the important distinction between our findings and the dogma associated with the natural timescale of degradation. We have also expanded on the discussion of our own results and their implications to gene response times. The specific changes are rather extensive and highlighted in red.

MINOR COMMENTS

1) The use of the word “network” in the Abstract could be misleading, as this creates the expectation that the work will deal with much general gene regulatory networks than the small motif analysed here. We suggest rephrasing and directly using the “single-input module” motif as introduced in line 13.

This is true, we have taken the referees advice and altered this statement.

2) Model: refer to Methods section when introducing the model, as well as in the Figure captions to point to where the rest of the parameter values are found (\gamma,\gamma_m_)

We now refer to the Materials and methods sections in Model as well as figure captions.

3) Notice typo on Fig. S1 (cel-cycle)

We have fixed the typo.

4) Fig S3 legend: Is it correct? The filled circles match the power law better than the open circles, whereas open circles are said to be auto-activation.
We have fixed the typo. The open circles represent auto-repression and filled circles auto-activation.

5) Fig. 5 Please revise the caption.

We have modified the caption to correctly read the panels.
