# Supplementary material for: Controlling gene expression timing through gene regulatory architecture
Source: PLoS Comput Biol. 2022 Jan 18;18(1):e1009745. doi: 10.1371/journal.pcbi.1009745 (PMC8797265; doi:10.1371/journal.pcbi.1009745)
Supplement: Attachment — Submitted filename: Referee_Response.pdf [file pcbi.1009745.s013.pdf]

We thank the reviewers and editors for the thoughtful assessment of our manuscript. We have amended the manuscript to address each specific comment from the referees below. We have updated Fig 1D to improve clarity. Changes are highlighted in red in the revised manuscript. Line numbers correspond to that from the revised manuscript.

Reviewer #1: In the revisions to "Controlling gene expression timing through gene regulatory architecture," Ali and Brewster focused on broadening the applicability and ultimately the impact of their work, appropriately addressing reviewer questions and adding thoughtful edits. I am happy to recommend acceptance of the manuscript.

The reviewers had challenged the authors with questions about the base assumptions of the model (decay rates), the relevance of the analyses (cross-regulating circuits), and the interpretations of the findings (what does the power law mean?). The authors satisfactorily addressed all points of clarity and adapted their model to answer the questions posed. They found new data to support their reasonings, and in a new set of supplementary figures, defended their work with an investigation of broader parameter sets and modeling schemes. This effort allowed them to draw broader conclusions about the relevance of this work across parameters that may be possessed by a more diverse set of organisms. Notably, these additional analyses and broadening of parameters did alter their conclusions.

While the authors have worked diligently to generate new supporting data to elevate the work, there are still a few lingering points (and minor edits) that the authors can address to further improve the manuscript.

1. While the authors have done a good job in improving the clarity and relevance of their conclusions with some added discussion of intermediate extrema and the interesting phenomena of "cross-regulated" targets, there are points at which the authors stop short of synthesizing the impact of their observations or addressing the limitations that prevent them from drawing such conclusions. Specifically, considering that the discovered power law properties have earned a full main text figure, it would be a lost opportunity not to comment on the impact of this phenomenon. It is understandable that the authors have not been able to pinpoint the underlying reason for this power law, but the mention of the power law without further comment in the added discussion paragraph (Lines 381-390 in the main text) leaves the readers questioning why this power law is mentioned at all.

In our view, in the absence of an underlying reason, the significance of the power law is that it gives us a "rule of thumb" on what to expect from changes in the system. For instance, Figure 3b tells us that we expect a TF with four times higher activation strengths to result in a doubling of the MFPT. The accompanying figure 3e tells us where this maxima is likely to occur in terms of binding affinity for the TF. Thank you for pointing out that we did not explicitly go over this in the discussion, we have added this to emphasize the usefulness of power-laws in gaining an intuition about complex systems..

2. Furthermore, the discussion section could still benefit from more clearly highlighting both the advantages and disadvantages of the presented model, placing it in the context of existing work. Related to the previous comment, if there are limitations of this model that prevent one from drawing precise conclusions, they should be mentioned in the discussion section.

We now mention the limitations of our model at the end of the Methods section.

3. Reviewer 1, specific comment #9 was not clearly communicated and seems to have been misunderstood. There is an inconsistency in the text likely stemming from a minor mix-up in the language. Currently, the manuscript reads “The steady state expression level decreases as the binding affinity is increased, from a maximal value of SSE=rb \/ γ_m γ for a very strong affinity when the promoter is always occupied by a TF to SSE=r_0 b \/ γ_m γ for very weak activation.” (Lines 156-159 in main text). However, both the plot in Figure 2C and the second half of that sentence conflict with the statement in the first part of that sentence. This should be corrected to either: “The steady state expression level decreases as k_off is increased…” or “The steady state expression level decreases as the binding affinity is decreased…” or some other variation.

For clarity, we have modified the sentence to read “ the steady state expression decreases as k_off is increased, from a maximal value of SSE = rb \/ γ_m γ for very strong activation ...” as suggested by the referee.

4. This was missed in the first round of revisions, but in the caption of Figure 6, the constitutive gene is said to be marked with “black asterisks”, but these appear rendered as filled-in small black circles.

Thank you for pointing it out. We have changed it to black filled circle.

5. Typo: line 321 “To further explore this relationship, In Fig. 6C…” should not have capitalized the word “In”.

We have fixed this typo.

Reviewer #2: The authors have addressed the main remarks of mi previous report. There are still a few points that in my opinion can be improved, in particular to improve the clarity of the manuscript and better integrate the outcomes of the model and simulations.

1. Authors: [...] Activating a promoter of a set rate will always make it reach a set level of expression faster than repressing it (in fact, it might not reach that level at all if you add repression). Using a relative threshold is a way to normalize two different scenarios to measure the dynamics of a promoter that reaches a set level of expression. As an example, the famous result that autorepression “speeds up” the response of a gene is only true if you take care to normalize the promoter such that the levels are the same (or to use a threshold).

I do understand that this is what it has been done in the literature, I was questioning about the biological relevance (since it should be the absolute TF concentration that matters, not the one relative to ss). This was however more a comment than a question, I do not know if the authors wish to discuss that, but I believe that it would help.

This is a conversation that we have in the lab with frequency and in the end we would say there is no “correct” answer but we do have a rationale for our choice in this study. For different proteins, different absolute numbers of them are impactful to the cell; some proteins may exist in the hundreds or thousands of copies to be effective while others may operate in extremely low copies (for instance DNA repair response via Ada: Uphoff *et al., Science* 2016). As a result, the idea we subscribe to here is that the gene is tuned to produce a relevant number of proteins at steady-state and thus we want to know the dynamics of response to approach that relevant number. However, we absolutely believe there are also situations and times when absolute concentration is the appropriate thing to consider (especially in terms of engineering applications).

2. Authors: [...] As for the origin of the power-law dependencies, we have spent considerable time on this and still don’t have a satisfactory explanation. We have tried to answer through toy models or exact solutions but have not had much success. [...]
I am a bit confused, and took me some time trying to understand. What about the new supplementary information S2 and Fig.S11? I know that this is not an exact solution, but it is still probably possible to improve our understanding from that. The SI should probably be valued more in the main text, and discussed. See also point below.

We now mention the results from the toy-model in the main text.

3. Authors: [...] The model can be solved exactly to get an analytical
expression for response time (time to reach a certain threshold, analogous to MFPT). The
analytical solution recapitulates all the features for an autotregulatory gene. However, we
could not get a simplified analytical form for peak response time, even for this simplified
model.

Again, reading the caption of S11: "(B) Peak MFPT versus alpha as well as koff at peak versus alpha (inset) from the toy-model matches well with the full deterministic model." This is not an analytical result but it shows that the toy model developed captures the power-law-like behaviour at least for the parameters chosen. I imagine it should then be possible to show that, with some approximations, by setting the derivative with respect k_off = 0 one obtains alpha^x. Otherwise, how the simulations compare to the toy model?

This was absolutely our first instinct and we thought this would be possible (taking a derivative and setting it equal to 0). However, unfortunately the optimization leads to a transcendental equation which we could not simplify to obtain peak MFPT or K_off as a pure function of other parameters, even with appropriate approximations. There may be a way to get to the solution through this approach, but we were not successful so we were limited to numerical validation.

It is (at least for me) very confusing (and time consuming) trying to understand the discussion on page 6, and the addition of $R = b r_0 + b(...$ is rather misleading. This approximation cannot infact hold as a function of time (it is assumed that n_TF = 1 at all times) and if I am not wrong, when computing the passage time to get to half of the SSE for a deterministic model dP/dt = R-\gamma P one would get the response time for a constitutive gene, \tau_cons in the SI.

We understand the confusion of the referee and apologize for not making it clear. We have rephrased the paragraph for clarity : Line 145-173.

We use *R*, the rate of production due to a single TF, as a proxy to gain an intuition on why we expect a non-monotonic behavior and not to get a quantitative answer. We must clarify that the steady state value is computed from the full deterministic model and then we use *R* as the rate of production to achieve the threshold. To be more precise, we use dP/dt = R(P) - \gamma P to compute the steady state (Yss) and use Yss / R(1) to compute the response time. This is absolutely only meant as a way to gain intuition about the non-monotonic behavior.

In fact, the referee is correct in pointing out that *R* is a function of time and we have explicitly mentioned this in the manuscript :

Ln 148: " the effective TF-production rate, on the other hand, has a complex dependence on time and changes as the number of TFs increases "

Instead of this part, I would suggest the authors to include here the results of the calculations done in the SI, and if/when possible, to better compare the results shown in Fig.3 to the model of the current SI.

We appreciate the suggestion of the referee. Now, we include the analytical expression of MFPT for the toy model in the manuscript.
